# Soft X-ray Laser Microscopy of Lipid Rafts towards GPCR-Based Drug Discovery Using Time-Resolved FRET Spectroscopy

**DOI:** 10.3390/ph4030524

**Published:** 2011-03-14

**Authors:** Motoyoshi Baba, Tohru Kozasa, Takao Hamakubo, Hiroto Kuroda, Kazuyuki Masuda, Shin Yoneya, Tatsuhiko Kodama

**Affiliations:** 1 Institute for Solid State Physics, University of Tokyo, Chiba 277, Japan; 2 Department of Ophthalmology and Advanced Laser Medical Center, Faculty of Medicine, Saitama Medical University, Saitama 350, Japan; E-Mails: kuroda@saitama-med.ac.jp (H.K.); shin@saitama-med.ac.jp (S.Y.); 3 Laboratory for System Biology and Medicine, Research Center for Advanced Science and Technology, University of Tokyo, Tokyo 153, Japan; E-Mails: kodama@lsbm.org (T.K.); hamakubo@lsbm.org (T.H.); masuda@lsbm.org (K.M.); 4 Department of Pharmacology, College of Medicine, University of Illinois, Chicago, Illinois 60612, USA; E-Mail: tkozas@uic.edu (T.K.)

**Keywords:** soft X-ray laser, soft X-ray microscopy, lipid raft, FRET, GPCR, drug discovery

## Abstract

Many signaling molecules involved in G protein-mediated signal transduction, which are present in the lipid rafts and believed to be controlled spatially and temporally, influence the potency and efficacy of neurotransmitter receptors and transporters. This has focus interest on lipid rafts and the notion that these microdomains acts as a kind of signaling platform and thus have an important role in the expression of membrane receptor-mediated signal transduction, cancer, immune responses, neurotransmission, viral infections and various other phenomena due to specific and efficient signaling according to extracellular stimuli. However, the real structure of lipid rafts has not been observed so far due to its small size and a lack of sufficiently sophisticated observation systems. A soft X-ray microscope using a coherent soft X-ray laser in the water window region (2.3–4.4 nm) should prove to be a most powerful tool to observe the dynamic structure of lipid rafts of several tens of nanometers in size in living cells. We have developed for the X-ray microscope a new compact soft X-ray laser using strongly induced plasma high harmonic resonance. We have also developed a time-resolved highly sensitive fluorescence resonance energy transfer (FRET) system and confirmed protein-protein interactions coupled with ligands. The simultaneous use of these new tools for observation of localization of G-protein coupled receptors (GPCRs) in rafts has become an important and optimum tool system to analyze the dynamics of signal transduction through rafts as signaling platform. New technology to visualize rafts is expected to lead to the understanding of those dynamics and innovative development of drug discovery that targets GPCRs localized in lipid rafts.

## 1. Introduction

Membrane or transmembrane receptors [1] are located at the cell membrane and play the very important role of signal transduction of the information from the outside into the cell. Physiologically active substances such as neurotransmitters, hormones, and autacoids transduce external information to the cell by binding with cellular communication receptors [2]. Secondary messengers from a receptor target proteins in a cascade and activate and transduce their information into the cell nucleus with very sophisticated controls [3]. The cell shows a unique response or a proliferation and differentiation reaction to such stimuli in accordance with the extracellular environment through various transgene expressions. There are three types of cell surface receptors:

1. Ion channel-coupled receptors [4];
2. Enzyme-coupled receptors [5];
3. G-Protein coupled receptor (GPCR) [6].

Many diseases are closely linked with the action of specific proteins, so the controller for a function of specific proteins is a molecular medicine [7]. GPCRs are proteins that have a very important role in the reactions of a living body by coupling to G-proteins [8]. GPCRs are the largest superfamily of proteins of extra seven-transmembrane segment receptors, which have a ligand binding site outside and a G protein coupling domain inside the cell [9].

There are more than 1,000 different members in a GPCR superfamily. Each member plays a very important role in controlling a reaction of a highly specific G protein due to their combination, and is greatly related to some disease in a living body. Therefore, GPCRs are widely considered the most important targets for drug discovery. In fact, according to their site of action nearly half of clinically used drugs can be assigned to this GPCR type [10]. Elucidation of signaling and regulatory mechanisms of transduction through GPCRs is a most important subject, and has been developed vigorously for the establishment of the molecular mechanisms of action or directions for the use of drugs in drug discovery [11].

A GPCR-related chemical genomics database is provided and information about GPCRs and their ligands are available from GLIDA [12]. Recently, genes of GPCRs with specific seven-fold repeats of a hydrophobic amino acid cluster could be directly found *in silico* by taking an effective step of sequential analysis of genomic DNA or cDNA finding a clue such as sequence, structure and mechanistic data about drug targets [13]. However, the ligands for those GPCRs found *in silico* are mostly unknown, so those GPCRs are called orphan receptors [14]. Only about 250 kinds of combinations of ligands and GPCRs became clear, and the other combinations of those GPCR and ligand pairs still remain unknown. Therefore, most of the physiological phenomena greatly responsive to GPCRs constitute a vast unexplored frontier for drug discovery. Many pharmaceutical agents on the market seek a target on receptors of widely covered fields such as a central nervous system, an endocrine system, a cardiovascular system, a respiratory system, a urinary system, a digestive system, a reproductive system, *etc.* We must devise strategies for the search for ligands combined with specific orphan receptors in the research to understand the regulation of physiological phenomena from the point of view of not only basic research, but also of applied research. Fierce competition for drug discovery in this field expands in the pharmaceutical industry and academia alike [15]. The concept of receptors or ion channels in considering their activities or effects of drugs were recognized by identifying substantial molecular mechanisms and functional significance. The investigation on the molecular mechanisms of how cells recognize the outside world has progressed remarkably in the 20th century [16]. It is recognized that there are various signal transduction pathways in the cell, such as secondary messengers through receptors or ion channels, a transcription factor, and a sequential gene activation control of the physiological cell activity. On the basis of this concept, we can approach a purposive drug discovery with signal molecules, molecular interactions, and signal transduction mechanism as the targets. To research the therapy for regulating a disease-causing biological activity by specifically controlling the cell information has become widely extended. In fact, most drugs used clinically all over the world have been successfully developed on the basis of this concept, and a further development of drug discovery in this way still continues vigorously. It is greatly expected that drug discovery will develop actively in cooperation with the various techniques of the cell informatics recognition mechanisms, therefore genome pharmaceuticals will strongly advance the drug discovery process and provide a stronger scientific basis for a specific genetic constitution in the functional genomics in the post-genomic era [17]. It has become to be recognized that cell ultrastructures of a few tens of nanometers in size (microdomains), which cannot be observed with an optical microscope nor with an electron microscope under living conditions, play a very important functional role in the molecular mechanism of biological reactions [18]. It has been proposed that these microdomains on the plasma membrane take a great part in the specific signal transduction through membrane receptors and the signal transduction mechanism of, for example, cancer, immune reaction,, viral infectious disease,, neurodegenerative disease, and cholesterol control [19]. GPCR signaling is not carried out uniformly on the cell membrane and has recently been shown to occur in microdomains called lipid rafts [20]. This mechanism of the regulation of GPCR signaling in lipid rafts is an interesting and important topic. The existence of lipid rafts on membranes has become widely recognized in various fields. The concept of signal transduction through a clustering of receptors and components of receptor-activated signaling cascades has become accepted, but its structure has not been elucidated due to its small size. The signaling molecules that cooperate with G-proteins located in lipid rafts are controlled spatially and temporally and influence the potency and efficacy of neurotransmitter receptors and transporters [21]. Lipid rafts perform the signal transduction of quick and efficient responses to stimulation, and also play an important role in the desensitization of signal transduction depending on the intensity of stimulation time and even the activation of another signaling system. The notion that these microdomains perform a type of signaling “platform” has become very focused on lipid rafts. The accumulation of evidence from various experiments has revealed the fact that they could function as a responsible transduction route [22]. Inside the cell, despite the presence of molecular groups related to the large volume of cell signaling, some of them respond quickly to various extracellular stimuli, and information for each has been translated into intracellular signaling, which will lead to the final cellular responses. Considering how quickly and orderly a flow of information is triggered and controlled, the idea that lipid rafts have a function as a signaling platform with a concentration of signaling molecules accumulated in the group there and specifically allow signaling for the extracellular stimuli efficiently came to be widely supported. Various approaches for visualization of the raft structure have been tried. Its size is currently believed to be on the order of tens of nm; therefore the resolution of a microscope using visible light is too low to observe lipid rafts.

Electron microscopy has adequate resolution to observe small samples of around 1 nm size, but requires pre-treatment or fixing of the samples. Due to strong tissue damage, the structure is not likely to maintain the rafts. Therefore, electron microscopy is not suitable for biological samples. As for the atomic force microscope (AFM) technique, the resolution has been enough to observe samples of tens of nm, but the sample structure changes due to the physical stimulus of the cantilever, and it takes time and effort to scan. An AFM is not a suitable instrument for the observation of the dynamic motion of a biomaterial [23]. Therefore, with the existing light sources and microscopes, it is very difficult to observe the raft structure, and it is necessary to develop a completely new approach. A soft X-ray microscope using a coherent soft X-ray source has a potential resolution of about 10 nm and can observe biological samples under wet conditions. This is why a soft X-ray microscope is the most likely candidate for imaging the lipid raft [24]. Furthermore, since the absorption of water is smaller compared to that of biological materials such as proteins, the spectral range with wavelengths from 2.4–4.3 nm (280–540 eV) region is called the water window. Within the water-window region, one should be able to directly observe living cells, as the effect of X-ray absorption by water can be small [25]. High-resolution observations of living cells or biological samples under wet conditions in the atmosphere could arise with the technological development of the analytical technology for single molecules in living cells, as well as the pathological analysis of the current advances in the reaction of external stimuli such as inflammatory cells and viral infections. An analytical technique to enable such a development has not been developed and will become an important key technology, leading to the elucidation of the molecular mechanisms of signal transduction. A new mechanism is expected to be a breakthrough in life sciences and a leading key technology to the development of new drugs. In the observation of the raft structure, it is desirable that the probe be specific for the configuration of membrane proteins and lipids. As mentioned above, rafts play an important role in various phenomena such as signaling, viral infections and immune responses. Using the integrated technical development of the probe, which specifically labels membrane proteins presented in a lipid raft structure, the development of novel therapies to elucidate the pathogenesis of cancer and lifestyle diseases and a detailed study of changes in quantitative and qualitative changes in signaling by molecular components of lipid rafts with aging and disease including even Alzheimer's disease, mad cow disease and other emerging infectious virus diseases with aging and disease become a variety of useful information for the treatment or prevention of disease. It is greatly expected to lead to the development of drugs that target the lipid rafts [26,27]. Thus, to observe the structure of a living cell with several tens of nanometers resolution, a soft X-ray microscope at the water-window wavelength region is a powerful tool. We discuss in this review the simultaneous measurements of visualization of lipid rafts and dynamics of the localization of GPCRs in lipid rafts using our new soft X-ray laser technique of harmonic resonance and highly sensitive time-resolved FRET spectroscopy.

## 2. Lipid Rafts and Signal Transduction

In 1972, Singer and Nicholson proposed the idea of fluid mosaic model whereby a phospholipid bilayer is a fluid form and the protein embedded in the membrane can move freely within it [28]. The types of lipids that configure the cell membrane are glycerophospholipids, sphingolipids, and many others. Moreover, there are many unsaturated carbon chains or lengths of alkyl chains in the lipid molecules of various types.

The outer and inner leaflets of the membrane have different lipid compositions. Whereas phosphatidylcholine, sphingomyelin and ganglioside are mainly present at the outer plasma membrane leaflet, phosphatidylserine, phosphatidylethanolamine and phosphatidylinositol are present in the leaves [29]. It has become apparent that small domains (microdomains) are formed on the cell membrane, from the observation that specific lipids such as cholesterol and glycosphingolipids separate from the other glycerophospholipid rich region based on their molecular characteristics [30]. For these microdomains, a variety of names have been used so far, and now the term lipid rafts is used collectively. The formation of these lipid rafts is thought to have been deeply involved in the molecular properties of those constituent lipids. Whereas phospholipids constitute a large proportion of the cell membrane and often have a side-chain unsaturated acyl chain, sphingolipids often take the form of a saturated acyl chain bound to a ceramide backbone. As a result, saturated acyl chains between sphingolipids are linear, and easy to work for the intermolecular interactions, so that the state has been taken as a tightly packed ordered liquid. On the other hand, phospholipids prevent a packing of lipid molecules due to the presence of the unsaturated acyl chains and takes on a disordered liquid state. Cholesterol is thought to support the linear carbon chains of lipid molecules and promote interactions between the lipids. A working hydrogen bond exists between the hydroxyl group of cholesterol and ceramide binding screens in the framework of sphingolipid. It is considered that this mechanism has contributed to the regular arrangement of cholesterol and sphingolipids [31]. In what is known as the old microdomains, caveolae (Latin for little caves), with sizes of 50–100 nm, were observed as flasklike recesses on the cell membrane [32]. Caveolae are considered to have relatively stable structures because they are backed by a caveolin protein (caveolin-1, -2, -3) in the cell membrane. Caveolin has a cholesterol binding activity, so caveolae has often been seen as a subtype of lipid rafts due to their richness of sphingolipids and cholesterol as well as lipid rafts. Note that all cells express a caveolin, but there are many cells that do not have caveolae. The molecules and composition of lipids localized in caveolae are not necessarily identical to those in lipid rafts. The possibility of the difference may also be considered by the function of caveolin [33]. Electron microscopic observations of caveolae were performed for a long time because of its morphological features. Many have been reported for localization of various molecules to caveolae by immune electron microscopy using colloidal gold-labeled antibodies [34]. Cold Triton X100 raft biochemical fractionation of cholesterol-enriched membrane fraction is recovered as a density light fraction with a density gradient centrifugation. In addition to the proteins such as caveolin and flotillin that are considered to be the so-called raft marker proteins, it is known this fraction has G-coupled receptor proteins, trimeric G proteins to conjugate, growth factor receptors such as EGF receptors and the secretase complex γ [35]. Since lipid rafts are those produced by the molecular characteristics of lipid, and there are different types of rafts due to the various lipid compositions, they always repeat the discrete set and are seen as a dynamic structure that changes size depending on the stimulus GPCR which are receptor membrane proteins that penetrate the cell membrane seven times and form a superfamily. Many receptors for neurotransmitters, hormones and autacoid belong to this superfamily. Currently, many of those drugs used clinically for the treatment of numerous diseases target the GPCR. It becomes very important to know the details of GPCR-mediated signaling. The analysis is being pursued vigorously [36]. Many of the GPCRs transduce information into the cell through the trimeric G protein. Gs, Gi, Gq, and G12 family are known as trimeric G proteins and there are several additional subtypes in each family. Due to the receptor activation, trimeric G protein separates into α subunit and βγ subunit. Gα subunit changes its state from GDP bound form (inactive form) to GTP bound form (activated) and dissociates from βγ subunit. Gα and Gβγ respectively will transmit a signal by controlling the activities of effector molecules such as enzymes or the channel activity [37].

Many molecules involved in signal transduction through trimeric G protein are known to exist in lipid rafts. We show cases that have been reported for localization to lipid rafts in Table 1, especially in basic molecular groups involved in signaling through trimeric G protein.

The mechanism of how such molecules localize on lipid rafts has been reported many times. Some molecules present in caveolae have a caveolin-binding motif. Among specific GPCRs existing in lipid rafts or caveolae, some receptors such as beta adrenoceptors and endothelin receptors have a caveolin binding motif too, and this mechanism that regulates the localization of receptors has been reported [38]. Many GPCRs have received post-translational glycosylation, and it is found that such glycosylation may have an association with lipid rafts in the case of sphingosine 1-phosphate type 1 receptors (S1P1) [39]. Trimeric G proteins have undergone post-translational modification by fatty acids, such as myristoylation and palmitoylation [40]. It is reported that these fatty acid modifications regulate the localization of trimeric G proteins to lipid rafts and G protein can be localized in caveolae through the direct coupling of caveolin [41]. In case of Gα12, it is reported G protein can be localized in caveolae through the direct coupling of heat shock protein 90 [42] Thus, it is becoming clear that GPCRs as bioactive receptors, trimeric G proteins associated with them, and the various effector molecules controlled by G proteins accumulate to the micro domains on the cell membrane called lipid rafts. From these evidences, lipid rafts are thought to be an informational field or platform, which act to convey the acceptance promptly into the cell after receiving extracellular stimuli. On the other hand, this fact suggests that the drugs that change the structure of lipid rafts may effect some modulation on the GPCR-mediated cell signal transduction [43]. A protein that joins the C terminal at the end of a GPCR has been considered to function as a factor that regulates subcellular localization of a GPCR. After agonists combine with a GPCR on the cell surface, GPCR-mediated signaling becomes activated. Therefore, GPCR translocation to the cell membrane surface is critical for the expression of biologically active, and those proteins that join C terminals of GPCRs carry on a part of regulation mechanism [44]. If some types of proteins that join specifically a GPCR are present, this system will impart a specific mechanism for the physiological function of the receptor expression.

**Figure 1:**
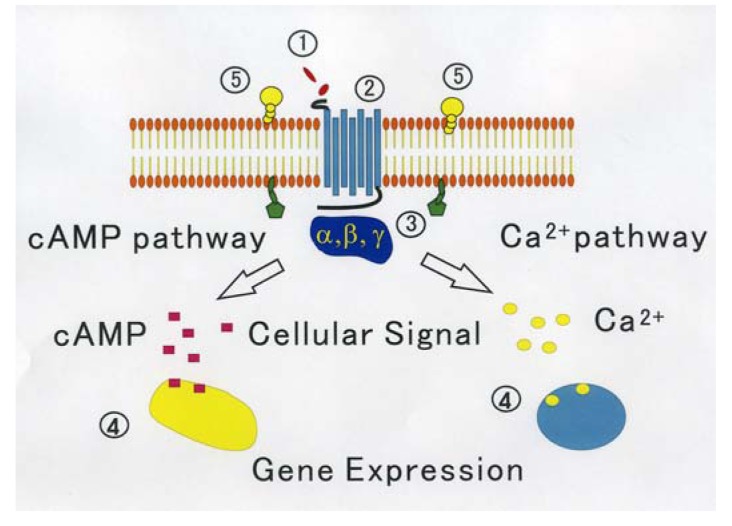
Signaling mechanisms of G protein-coupled receptors proposed as a second messenger theory (1). Stimuli from the outside world, such as ligands, agonists, antagonists, autacoid with different chemical structure; (2). GPCR has the extracellular ligand binding sites and intracellular G protein binding domains. Ligands *etc.* couple a seven transmembrane receptor and G protein is activated through a GPCR; (3). There are four major groups of trimeric G proteins such as Gi, Gq, G12, Gs. They are first targets of GPCR-mediated signal; (4). By the action of activated G proteins, depending on the type of G protein, e.g., cAMP and calcium ion concentration in cells change. The gene expression is regulated by phosphorylation in cells propagated as more; (5). GPI (Glycosylphosphatidylinositol)-anchored protein. During signaling, many GPCRs are integrated into lipid rafts. Lipid raft is a specific microdomain of cell membranes and acts as a signaling platform for GPCR signaling.

On the other hand, through analyzing in detail the biological activity of the protein joined the C terminal of GPCR that affects the function of GPCR, a new activity regulation mechanism of GPCR becomes clear. These proteins are considered to be new target molecules that regulate a function of GPCRs [45]. We show the diagram of various regulatory mechanisms of GPCR in Figure 1.

Thus lipid rafts have been reported to be involved in the pathophysiology and a variety of physiological functional expressions such as intracellular vesicular transport, cholesterol transport, neurite outgrowth and immune responses [46]. Recently, neuraminidase which has its own fate for neuronal axons of nerve cells (sialidase: ganglioside sialic acid cleavage enzyme) was reported to be involved in activity. It has been suggested that configurational changes of lipid rafts impact nerve function [47]. On the other hand, due to the relationship between pathology and expression of lipid rafts, it is well known that lipid rafts are used on the path when viruses such as influenza and HIV and others or bacteria invade a host cell. It is known that bacterial toxins bind to cells via gangliosides as a scaffold and work. For example, cholera toxin binds to GM1 ganglioside, and tetanus toxin and botulinum toxin bind to GT1b and others have been reported [48]. Aggregation and sinking of amyloid β peptide in Alzheimer's disease can occur at the GM1- or cholesterol-rich lipid rafts on the cell membrane of nerve cells as a scaffold has been reported [49]. The relationship between prion diseases and lipid rafts has also been reported [50]. There are many reports about the relationship between lipid rafts and disease expression, as mentioned above. In addition, an insulin receptor is present in lipid raft/caveolae fractions as one of the tyrosine kinase-type receptors, which acts as if it were the scaffold needed when information is transmitted from activated insulin receptor to the next signaling molecule [51]. As another example, following the antigen-specific receptor TCR (T cell receptor) presented on the surface of T cells receives activation signals, a large raft aggregation of lipid rafts is formed accompanied by a number of signaling molecules. A variety of signaling cascades proceeds at once [52]. Thus the relationship between a number of signaling and lipid rafts has been pointed out. The knowledge of the concentration of signaling-related substances in the lipid rafts is being accumulated.

## 3. Research and Discussion

### 3.1. Visualization of Lipid Rafts and Time Resolved FRET Spectroscopy

Since a membrane raft structure cannot be seen as a fine image, there still exist some researchers who question the substance of rafts, although rafts are clearly defined biochemically and the recognition of a raft substance has become an important concept in understanding the pathogenesis of diseases such as Alzheimer's disease, transmission mechanism of receptors and viral infection mechanisms. There has been increasing awareness of its importance even in fields such as neuroscience or immunology. Even now, there are no instruments that can image raft structure reliably, because the size of a raft is too small to observe with an optical microscope, and furthermore it its structure changes dynamically. Under such circumstances, from the point of view of not only academic significance but practicality and usefulness, the development of a technology to visualize a raft is a very important applied technology for pathogenesis and novel therapeutic development of cancer and lifestyle diseases, as well as diseases such as Alzheimer's, mad cow disease and infectious emerging viruses.

Detailed examination of quantitative and qualitative changes in signaling by lipid rafts along with other molecular components due to disease or aging is very useful information for the prevention and treatment of disease, and much is expected of drug development that targets GPCRs localized in the rafts. We have developed a new X-ray laser using plasma harmonic resonance [53]. Visualization of lipid rafts with this new light source and using sensitive time-resolved high resolution time resolved Fluorescence Resonance Energy Transfer (FRET) spectroscopy to observe the expression of protein function simultaneously, which has been done at first by us [54], will reveal the real dynamics of the proteins and we will be able to discuss the localization of GPCRs on the rafts and signal transduction through them. The dynamics of the intracellular receptors, in particular the localization or transportation, show a rapidly evolving landscape in the future studies on molecular mechanisms. That is, for the coupled receptor function and drug affinity, G proteins were hitherto highlighted by the differences of the type. Localization of protein receptors is being recognized as a critical factor in the cell information [55]. In fact, judging from the biological control mechanisms, trying to achieve a variety of communication materials for a limited operation predicts that the factors such as the localization in cells is very important. Through more detailed analysis of intracellular localization or regulation of the receptor and the relationship between disease and drug action, the new concept of regulation of cellular receptors may be expected to become clear.

### 3.2. Observation of Rafts Using a New Soft X-ray Laser

Visualization of the raft structure has been tried using various approaches, but rafts cannot be visualized using an optical microscope due to its poor resolution. Electron microscopy needs pre-treatment of the sample under dry conditions. Therefore, the raft structure is not kept in a fixed condition and thus should be observed by a whole new method.

The soft-X-ray wavelength region of 2.3–4.4 nm (280–540 eV) is called the water window, because of the small absorption by water compared to other biomaterial such as lipids or proteins, and this area has been largely left unexplored in science and technology. Many researchers around the world are competing in the research and development of X-ray lasers, which is expected to have large impact on the application fields of molecular biology, life sciences and semiconductor engineering. The smallest observable size is limited to the wavelength of the light source used. It is well known that if the light beam is coherent, this resolution becomes 0.61λ (λ: wavelength) [56]. Therefore, when we observe small particles such as rafts, the light wavelength must be shorter than the size of the object.

**Figure 2:**
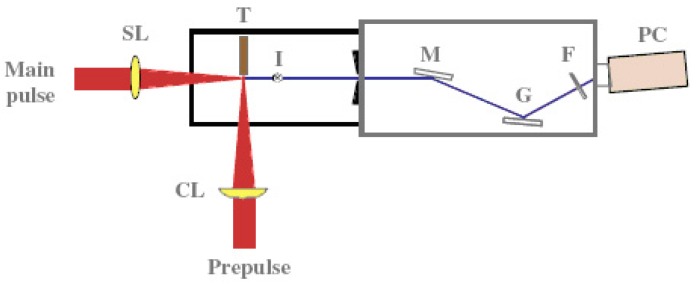
Diagram of the experimental setup. The symbols shown in the figure correspond to spherical lens (SL), cylindrical lens (CL), molybdenum target (T), position that is spatially imaged onto the spectrometer focal plane (I), cylindrical gold-coated mirror (M), Hitachi flat-field grating (G), 0.65 μm thick Al foil (F), and photocathode camera (PC).

**Figure 3:**
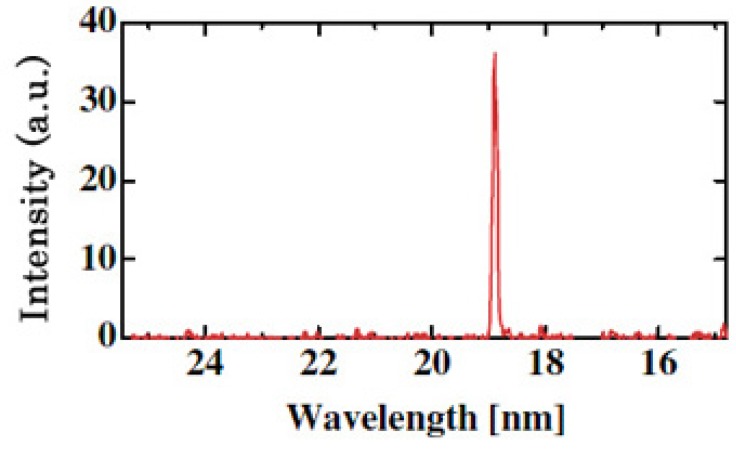
Intensity trace of the on-axis soft-X-ray spectrum between 15 and 25 nm is shown. The transient collisional excitation (TCE) X-ray laser has been successfully demonstrated. We observed highly directive 18.9 nm nickel-like molybdenum X-ray laser operation.

A coherent single-wavelength laser within the water-window region has long been viewed as a dream instrument, and has been developed under keen competition in many countries such as United States, Germany, Canada France and China [57-59]. Realization of a small and practical short wavelength soft X-ray laser will become a breakthrough for the research field, which is expected to be applied extensively and practically. Its arrival will result in a significant impact of expanding large development in creation of emerging interdisciplinary research. We have proposed and successfully operated an original new scheme of a longitudinal transient collisional excitation (TCE) soft X-ray laser [60]. The experimental diagram is shown in Figure 2. We show the obtained Ni-like Mo coherent soft X-ray spectral line at 18.9 nm in Figure 3. The Ni-like silver soft X-ray laser at 13.9 nm [61] and the Ni-like La soft X-ray amplification at 8.8 nm [62] were reported using this TCE scheme. We can reach the water window at 4.3 nm using a W (tungsten metal) target in this way. However, the energy needed for excitation increases proportional to λ^−4^ (λ: laser wavelength), so the equipment becomes large, a little complicated and it costs too much.

**Figure 4:**
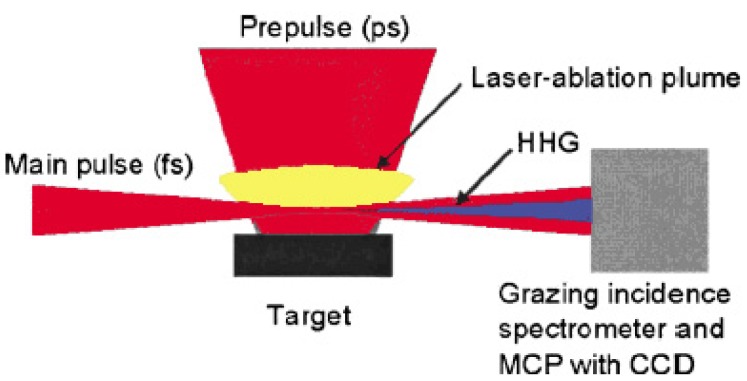
Experimental setup. The prepulse was focused on the target surface and, after 88 ns, the femtosecond laser pulse was focused onto the laser-ablation plume. The generated harmonics (HHG: high-order harmonic generation) were measured by using the grazing incidence spectrometer with the MCP (micro-channel plate) and CCD (charge coupled device) camera.

The wavelength of high harmonic generation has reached 7.9 nm in the short wavelength region, which is the 101st harmonic of the 800 nm fundamental Ti:sapphire laser light [63]. We also observed for the first time a strong and efficient laser induced resonance harmonic using laser-ablated plasma. Figure 4 shows the schematic diagram of our laser induced strong resonance harmonic generator of coherent soft X-ray.

**Figure 5:**
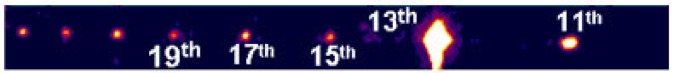
Strong resonance enhancement of a single harmonic is generated from the interaction of a femtosecond pulse with low ionized indium ablation. 4d^10^5s^2^.

**Figure 6:**
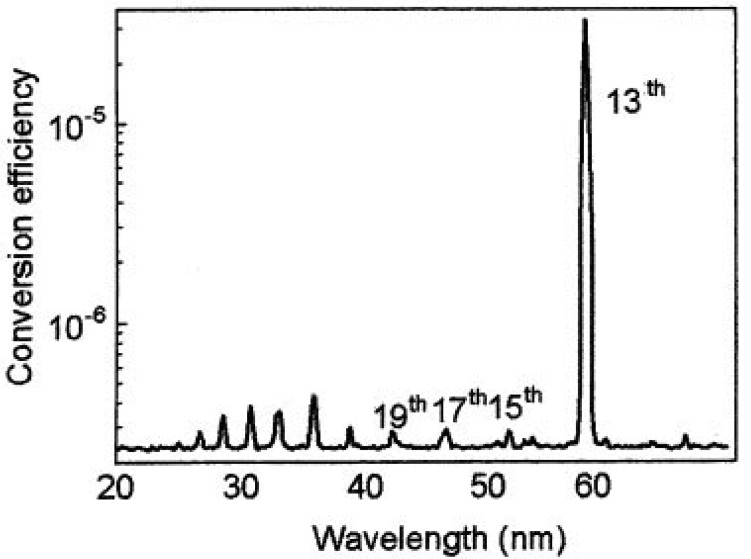
Spectrum of the high-order harmonic generation was obtained using the indium plasma. The pulse energy of the 13th harmonic is about 1 μJ.

Typical coherent laser induced plasma high harmonic using In (indium) is shown in Figure 5 and Figure 6. This new light source is a quasi monochromatic and coherent soft X-ray source, which has an extraordinary performance of ultra-high efficiency and high directivity. Those lines are the 13th (61 nm) via In (indium), 17th (46.8 nm) via Sn (tin), 21st (37.8 nm) via Sb (antimony), 27th (29.4 nm) via Te (tellurium) [64].

**Figure 7:**
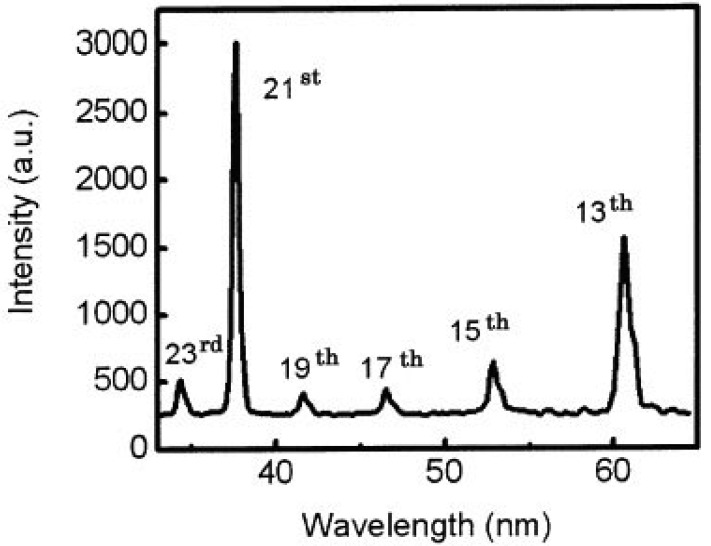
An intense exact resonance enhancement of single high-harmonic from an antimony ion by using Ti:sapphire laser at 37 nm was obtained. The intensity of the 21st harmonic was 10 times higher than those of the 23rd and the 19th harmonics.

We also got two simultaneous high harmonics such as 13th and 21st using double target, as shown in Figure 7 [65].

This new light source is a monochromatic and coherent soft X-ray source, which has an extraordinary performance of ultra-high efficiency and high directivity. Its conversion efficiency is about 10^−4^, which is much higher compared to that obtained by similar techniques with a gas jet. One laser pulse has more than 1 micro-joule of energy, which is more than 1,000 times stronger than those light pulses from monochromatic synchrotron radiation light source. Our new laser system combines a number of excellent performances such as high-intensity, short pulse (femtosecond or attosecond time scale), high spatial coherence, high repetition and compactness. This advanced laser system is suitable for various applications ranging from biotechnology to the development of nanomaterials and is particularly the best light source to observe lipid rafts. Soft X-ray microscopy using a plasma harmonic generation source and the coherent X-ray diffraction imaging will dramatically heighten the spatial resolution, which enables one to observe raw wet materials such as the raft structure. No one has ever seen the lipid rafts in the living cell. Visualization of those rafts is dreamt of among medical scientists and biologists, and it will lead to the development of innovative new therapies for various infectious diseases. Thus, the concept of lipid rafts are very important to elucidate the virus infection mechanism and the pathogenesis of Alzheimer's disease, and observation of the lipid rafts will have a tremendous and significant impact on innovative new therapies. The development of a single molecule imaging of three-dimensional GPCR signaling leads to entirely new insight, and this is a breakthrough technology leading to new drug discovery mediated GPCR transport system.

### 3.3. Time Resolved FRET Spectroscopy

Fluorescence spectroscopy, especially in combination with laser techniques is a very powerful tool and has a very high sensitivity and enables us to measure a weak signal such as the single photon counting [66-68]. It can be used to detect or trace an infinitesimal quantity of material and fluorescence properties (spectral intensity) can serve as probes for changes in the microenvironment. In particular, fluorescence imaging technique is a powerful tool in the pharmaceutical industry for drug screening. Visualization of live cells using gene technology is a revolutionary new technology, and has a great impact on life sciences and brings about a new phase of research. FRET is a mechanism whereby fluorescence energy is transferred before emission from one molecule to another within the Forster distance of about 1–10 nm, if there is an overlap of spectra between the emission of the first molecule (donor) and absorption of another molecule (acceptor) [69,70]. This FRET technology is a spectroscopic technique used in drug discovery industry, especially, as an important tool for analyzing the interaction between GPCRs and ligands.

Shimomura discovered and isolated a wild type GFP (Green Fluorescent Protein) from *Aequorea victoria* (luminescent jellyfish) and was awarded the Nobel Prize in 2008 for this distinguished and meritorious discovery. GFP is constructed from more than two hundred amino-acid sequences. Three of the amino acids included in the GFP contribute to the dye part as a consequence of condensation reaction and oxidation reaction. Whereas a normal fluorescent protein molecule is composed of two parts, *i.e.*, one is a protein and the other is a fluorescent molecule, a GFP has a molecular structure that includes a fluorescent chromophore in the protein itself. Therefore, a GFP in the whole is one molecular protein and the chromophore of it is a part of a GFP protein. From this fact, GFP was found to have the possibility of gene cloning. In 1979, this important discovery of the structure of GFP lead to the epoch-making technology of the expression of green fluorescent protein molecules by introducing a GFP gene clone into living cells with a genetic technology. In 1992, Prasher made a GFP clone and Chalfie successfully showed the expression of GFP in living organisms in 1994 [71]. With the great contribution of the genetic engineering technique, we can introduce fluorescent molecules freely into cells without the need of genetic products derived from a jellyfish, and obtain emitting light without being influenced by the species expressed. Fluorescence proteins have played an important role in gene expression as a marker for fluorescence microscopy by the expression of the gene cloning and heterologous systems [72]. Tsien *et al.* have also made fluorescent proteins of different fluorescence colors in adding additional various improvements for GFP proteins [73]. There are several mutant GFP chromophores created by substitution of some amino acids, such as BFP (Blue Fluorescent Protein), CFP (Cyan Fluorescent Protein), ECFP (Enhanced Cyan Emitting GFP), YFP (Yellow Fluorescent Protein), and EYFP (Enhanced Yellow Emitting GFP). There are differences in emission and absorption properties between them.

**Figure 8:**
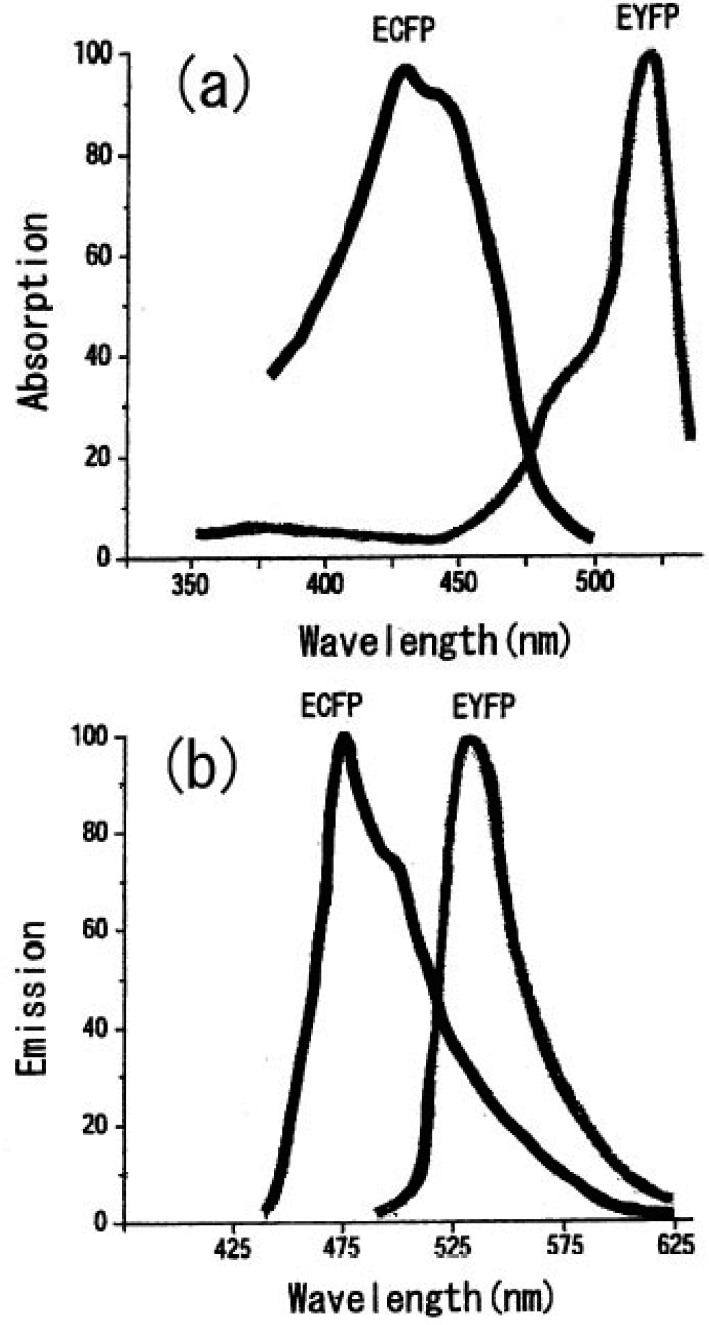
**(a)** Absorption and **(b)** emission spectra of various GFP mutants.

We can select the donor-acceptor pair proteins among these fluorescence proteins, which cause an efficient FRET function. For example, the CFP and YFP pair is a good candidate for FRET spectroscopy as shown in Figure 8.

**Figure 9:**
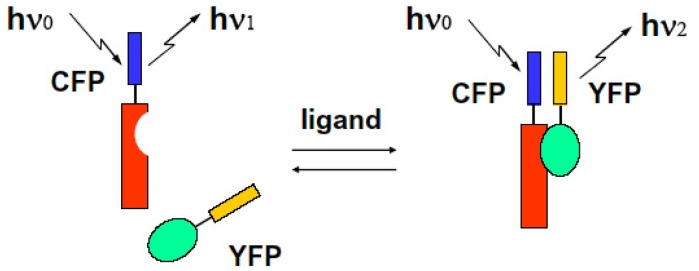
FRET phenomenon occurred between hetero-molecules.

**Figure 10:**
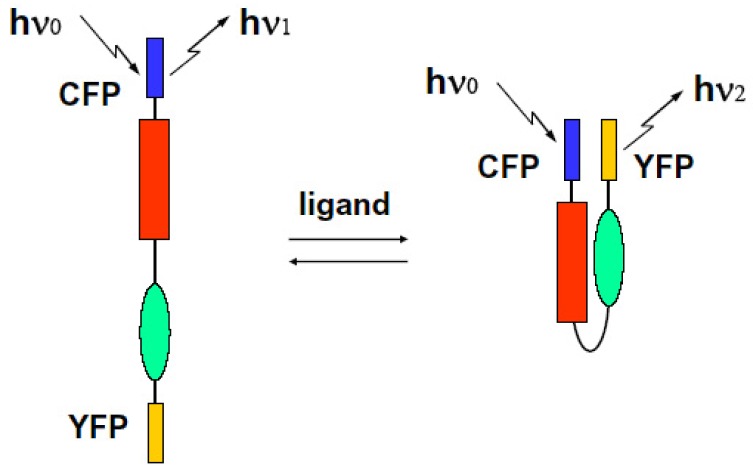
FRET phenomenon occurring intramolecularly.

We can observe the FRET signal of an interaction between two different proteins attached with CFP or YFP, respectively (two molecular system), or the structural change of molecule due to ion-sensitive as a FRET signal under the condition on that both ends of an observed molecule are attached with different GFPs (one molecular system). We show these schematic diagrams in Figure 9 and Figure 10.

FRET is the response of the dynamic motion of proteins at close range. That is why we can recognize in which steps the kinesin adopts a one-headbound intermediate or both heads are bound to adjacent tubulin subunits [74]. Optical marking technology of living cells has become a tool that cannot be neglected in essential medical and biological research. It is used mainly in fields such as cell lineage, the conversion of large cell position, movement, the study of embryology and morphology of the formation of functional networks, regenerative medicine and neuroscience. Understanding the spatial and temporal control of signal transduction with co-operation of FRET system and a soft X-ray microscope to observe dynamic changes such as position and movement in living cells leads to understanding of the pathogenetic mechanism of diseases such as cancer and immune responses. At the same time, it will have a very important role for ultra-sensitive HTS (High-throughput screening) of new drugs. With a conventional one molecular FRET technique under steady state excitation, Miyawaki *et al.* developed fluorescent indicators for calcium ions when bradykinin was administered in astrocytes expressed cameleon. Cameleon protein consists of tandem fusions of a blue- or cyan-emitting mutant of the GFP, calmodulin, the calmodulin-binding peptide, and an enhanced yellow-emitting GFP [75]. Cameleon is sensitive to calcium ions. If administration of calcium ions should cause the structural changes of closing both protein molecules as a bending result, when we selectively excite CFP at appropriate wavelength of light, excitation energy transfers from CFP to YFP with some probability due to the overlap between emission spectra of CFP and absorption spectra of YFP. As a result, even if an excitation is performed at non-absorbable region, a YFP should emit fluorescence using energy received from CFP emission, and emission intensity from a donor CFP itself decreases because of giving its energy to YFP. Under steady state excitation condition, we can observe the FRET phenomenon as a change in the ratio between the CFP and YFP emission intensities at a non-overlap wavelength region. Localized Ca^2+^ signals and protein heterodimerization in individual live cells were observed by using conventional FRET technique [76].

By the way, a conventional FRET spectroscopy can reveal the structure change only for the case ion which protein is equally attached with CFP and YFP, such as cameleon. In the case that acceptor fluorescence proteins are added later (*i.e.*, two molecule systems), spectral distribution changes naturally, so we cannot use this technique to observe the interaction between proteins. Thus FRET spectroscopy is a non-destructive method and can inform us about the interactions between fluorescence proteins or structure changes under steady state condition, but there has been no direct observation of real time nanosecond resolved ultra fast FRET dynamics in proteins. We developed an ultra fast time-resolved system to get a signal as a decay time change of fluorescence due to FRET. If we can directly observe the time-resolved spectral features in protein interactions, we can directly examine protein modification and the interaction or dynamics of ion-sensitivity occurring during ligand binding. Using this ultra fast time resolved FRET spectroscopy system, we can measure the decay time shortening of fluorescence as a result of the interaction between donor-acceptor pair proteins. We can also measure a spectral distribution change of fluorescence at the initial stage of the FRET mechanism and confirm the dynamics of the accessing between proteins on the ligand induced activated state. We define the rate constants of fluorescent energy transfer and donor emission k_T_, k_f_. They are related as follows [77]:

(1) $$k_{T}=\left\{\frac{9000*\ln10}{128\pi^{5}n^{4}N_{A}}*\frac{\kappa^{2}}{R^{6}}*J\right\}k_{t}$$

(2) $$J=\int {ɛ}_{A}\left(\lambda \right)F_{D}\left(\lambda \right)\lambda^{4}d\lambda /\int F_{D}\left(\lambda \right)d\lambda$$

where n is index of solvent, N_A_ is Avogadro's number, κ^2^ is the orientation factor of overlapping for the donor and acceptor transition dipole moments, R is the distance between two molecules, ε is the acceptor absorption coefficient, F is emission spectrum distribution, λ is wavelength, *J* is the index of resonance energy overlapping for donor emission spectrum and acceptor absorption spectrum. The rate constants of fluorescent energy transfer k_T_ decreases proportional to the inverse six powers of ten, so we can sensitively detect the distance between interaction molecules. After the excited donor interacts the acceptor molecule, the decay time of emission from the excited donor becomes:

(3) $$\tau /\tau^{\prime }=1+k_{T}\tau$$

where τ' is the decay time of fluorescence from an excited state donor with an acceptor, τ is without an acceptor. This shows the lifetime of the fluorescence of a donor shortens by transferring its energy to an acceptor. Thus we can directly investigate a ligand-induced deformation of proteins, an interaction between proteins on ligand binding, and ion sensitivity. This system is also notable as an important means to observe changes in molecular structure and binding, the dynamics of dissociation and activation state [78]. Protein interactions can be directly observed as time-resolved spectral features in this way. This system enables us to construct an up-to-date highly sensitive detection for signal transmittance of luminous viruses in fusion with activators of G protein on nano-sized biochips not in cells. This system is also expected to play a major role as the probe, because measurement is able to trace a small amount and fluorescent properties are highly sensitive to the environment and state of measured molecules.

**Figure 11:**
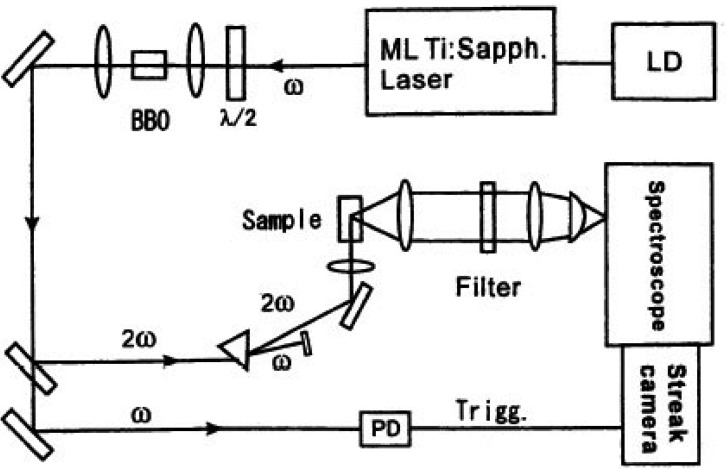
The schematic diagram of ultra fast time resolved FRET measurement system composed of a mode locked Ti:sapphier laser (ML Ti:Sapph.Laser), which was excited with a power controlled diode laser (LD) and a streak camera with a monochromator. Excitation laser pulses are extracted with the electro-optic modulator. A frequency doubled laser beam was generated using a nonlinear BBO (beta barium borate: β BaB_2_O_4_) crystal. Fluorescence from luminous protein excited with a frequency doubled laser beam is detected and stored two dimensionally (temporal, spectral) using a photon counting Streak camera system.

According to this approach we measured the decay time shortening of fluorescence. The sample was excited with an ultra short laser pulse from a high repetition mode-locked laser. Single-photon counting measurement was done for emission from a fluorescent protein to have spectral and time-resolved data. We show the experimental setup of the observation system in Figure 11.

We excited the donor fluorescent protein spectrally selective with second harmonic of the high repetition rate ultra short mode-locked Ti:sapphire laser excited by second harmonic of Nd:YVO_4_ laser (Spectra Physics co., model Tsunami, wavelength: 800 nm, repetition rate: 82 MHz, pulse duration: 100 femtoseconds). If necessary, laser pulse trains were extracted with the E.O. modulator (Conoptic co., Model 350) and this extraction guarantees the absence of any entanglement effects of multi fluorescence components. The delay time from the excited laser pulses for each fluorescence photon of light emitting proteins was measured with a monochromator and the streak camera (Hamamatsu Photonics co., model C4334, time resolution is 10 ps) using a photon counting method. The observed counting data were integrated as two-dimensional data of delay time and fluorescence wavelength spectrum. We can proceed the decay time of spectrally resolved fluorescence from luminous proteins in a specific wavelength region and compare the spectral distribution as a temporal stack of the luminescence with the emission ratio spectral data by a conventional constant excitation method. We measured the time-resolved fluorescence from a cameleon and an ECFP-GRIN (G protein-regulated inducer of neurite outgrowth) protein pair with this new technique for the first time [54]. A GoVenus protein is a protein fusioned with a YFP into Gαo, which couples with the G protein-coupled receptors. A Grin protein specifically interacts with a GoVenus and an activated Gαo (GTP form). We used an ECFP-GRIN protein fusioned with a CFP at the interaction port of Go. An activated Gαo deforms into a GTP form and makes a complex with a GRIN protein in a way to get into the protein binding.

Kozasa *et al.* proposed the concept of the regulators of G protein signaling (RGS) and discovered GRIN proteins. GRIN is a novel RGS protein expressed largely in brain and binds specifically to an activated Gαo protein (GTP form) through its carboxyl-terminal region [76]. Thus a GRIN-GoVenus protein pair is a good candidate for donor-acceptor protein pair for the FRET system. GRIN protein family are novel membrane-bound proteins and candidates for G protein effectors, which are identified by screening a cDNA expression library with phosphorylated GTPγS-Gzα as a probe of very low concentrations required. Both GRIN1 (Z-16) and GRIN2 (KIAA0514) bind to activated Goα through its carboxyl-terminal region. Coexpression of GRIN1 or GRIN2 with activated Goα causes formation of a network of fine processes in Neuro2a cells, suggesting that these pathways may function downstream of Goα to control growth of neurites [79]. When a ligand combines specifically with a GPCR and it stimulates heterotrimeric G proteins (Gα, Gβ, Gγ) to separate Gα and Gβγ. An activated Gα reconstitutes to combine with an effecter protein (in this case *i.e.* GRIN). We added AlF4- ions and stimulated Goα to make the activated GTP form. We confirmed the interaction between ECFP-GRIN (CFP) and activated GoVenus protein (YFP) from the change in fluorescence lifetime and spectral distribution using processing data of FRET signal. ECFP-GRIN proteins (donor) were excited spectrally selective (CFP: ex.434 nm, em. 474 nm, YFP: ex. 514 nm, em. 527 nm) and emission from GoVenus (acceptor) was detected as the spectrally resolved delay time from the excited laser pulses for each fluorescent photon within 10 ns with the streak camera (Hamamatsu Photonics Co., model C4334, time resolution is 10 ps) under conditions of activated (FRET) or non-activated (non-FRET) state. We counted photons that came to each channel of the streak camera. Those photons obtained as a fast component were derived from a pure FRET effect. Those accumulated data are plotted in a spectrogram. We measured the CFP or YFP fluorescence intensity concerning the FRET phenomena by integrating the photon counts between the spectral window specific for CFP protein or YFP protein.

**Figure 12:**
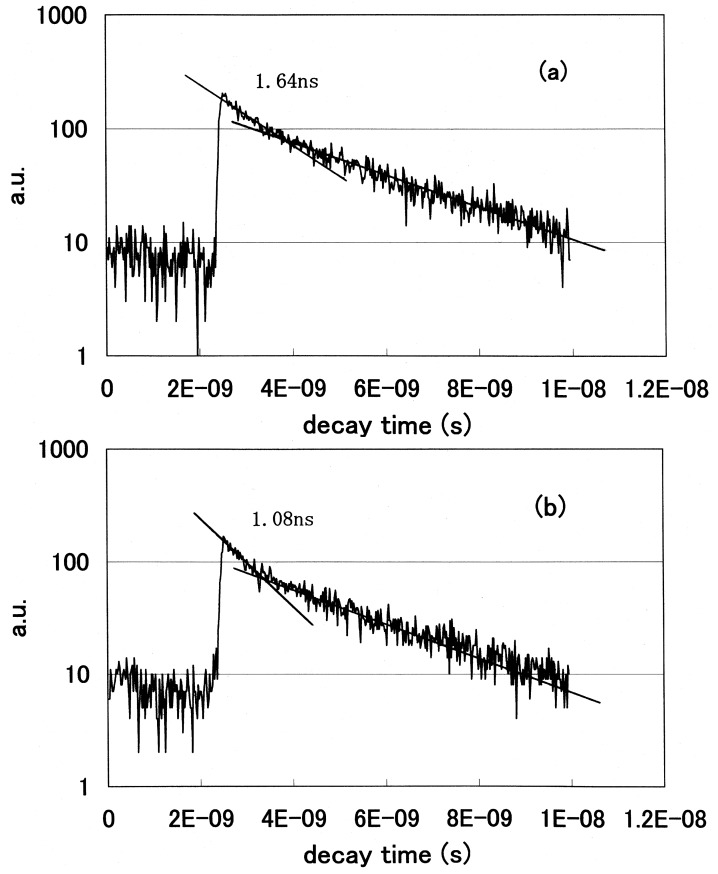
The decay curves of fluorescence from a GRIN1-GoVenus protein pair in the (**a**). non-activated and (**b**). activated state.

We statistically determined the decay time of fluorescence at each wavelength and acquired the spectral distribution as a temporal stack of the luminescence emitted at the initial stage of FRET. We plotted the decay curves of fluorescence using a GRIN-GoVenus protein pair system. We counted photons in the 465–500 nm wavelength region, where a donor CFP emits but an acceptor YFP does not emit. We show the decay curves of fluorescence from GRIN-GoVenus protein pair system in Figure 12(a) under the condition of non-activated (non-FRET) and (b) an activated (FRET) state. There are fast and slow components in the fluorescence decay curve of a donor protein using our time-resolved FRET system. To confirm which component is originally derived from fluorescence energy transfer from a donor to an acceptor, we extracted laser pulses using an electro optic modulator to extend the repetition rate and fitted the data multi-exponentially. The fast component drastically changed, but the long component did not change its decay time. From these results, only the fast component is derived from the FRET mechanism. Since the decay time of a fast component is about 1 ns, we opened the measurement window of the streak camera to 10 ns to detect photons coming from luminous proteins after every excitation pulse and closed the gate before subsequent photons came. The laser oscillation repetition rate was 82 MHz, *i.e.* every 12 ns.

We clearly observed the drastic shortening of donor fluorescence decay time in the FRET condition for both protein pair systems. Both decay curves showed multi-exponential decay. We fitted the data multi-exponentially and calculated the decay time. We showed the fast and slow components of decay times of the donor CFP fluorescence in Table 2.

**Figure 13:**
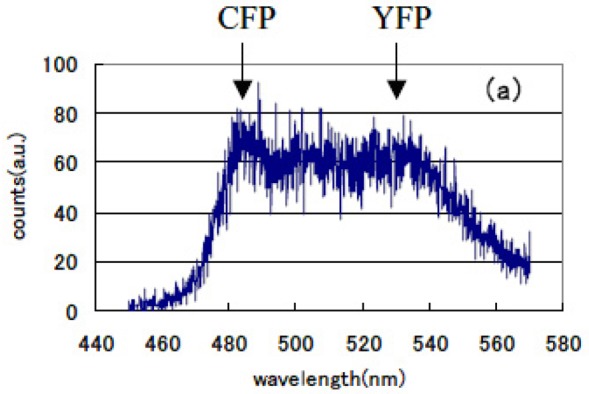

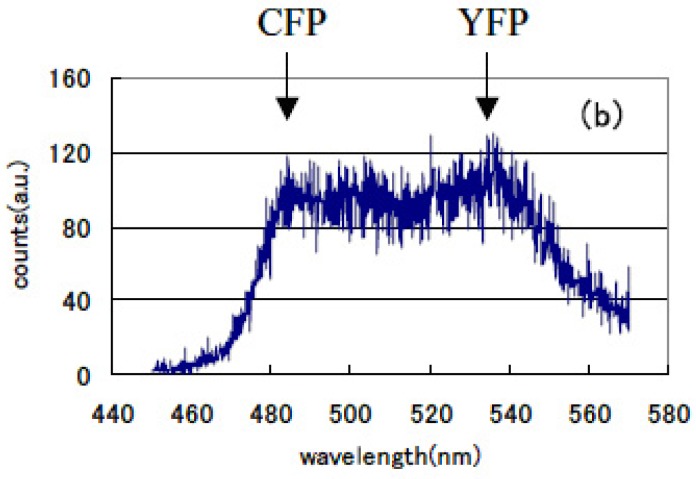
Emission distribution of spectrum from cameleon in the (**a**). non-activated and (**b**). FRET condition using this time resolved FRET system. Fluorescence from CFP and YFP protein is shown by the arrows.

The lifetime of cameleon-CFP fluorescence shortened from 1.1 ns to 0.9 ns (18% decrease). In the case of GRIN-GoVenus protein pair system, the lifetime of effecter GRIN2-CFP fluorescence drastically shortened from 1.15 ns to 0.6 ns (48% decrease) and for GRIN1 fluorescence lifetime shortened from 1.26 ns to 0.8 ns (36% decrease). In this study, we can identify the fluorescence lifetime of CFP around one nanosecond and got the integrated spectrum of only fast decay component of FRET signal at first. This system enables us to trace the dynamics of the interaction between proteins at the ligand induced activated state, molecular structure change and combination or dissociation and will be one of a key technology for the bio-chip sensor technology. Binding of calcium ions to a cameleon protein makes calmodulin wrap around the M13 domain and CFP contacts and transfers its energy to YFP. An increase of the fluorescence resonance energy transfer between the flanking GFPs changes spectral distribution of fluorescence from luminous proteins. We added the data stored within 10 ns from excitation as temporal stack of the luminescence at every wavelength channel and acquired the emitted spectral distribution. Figure 13 shows the spectral change of fluorescence from the bent cameleon induced by with [Figure 13(a)] and without [Figure 13(b)] calcium ions. This data is acquired time-resolvedly at the initial stage in the FRET condition.

We recognized the increase in the ratio between CFP and YFP peak emission intensities (shown by the arrows in the Figures) was about 29% in the FRET condition after adding calcium ions compared with that for the non-activated condition. Increase in the ratio of their emission intensities under constant excitation was reported by Miyawaki *et al.* [75]. We compared these time-resolved spectral emission distribution data to those obtained using conventional stationary excitation. They show a good coincidence with the time-resolved spectral data acquired in our new experimental method. We confirmed that fluorescence resonance energy transfer has already happened in a very short time of 10 ns immediately following the excitation pulse seeing that the spectrum changes according to our time-resolved FRET spectroscopy. We can easily compare these emission spectral distribution data and those observed by a conventional stationary excitation. Both showed good correspondence.

## 4. Conclusions

So-called dry biology has approached the structural analysis of membrane proteins, but there was a problem of fixation or biological injury. These issues are resolved due to coherent soft X-ray microscopy of living cells by wet approaches using soft X-ray laser sources. The development of a soft X-ray microscope is not limited to imaging of rafts and would have significant effects on the contributions to the field of transcription regulation which is the most important research in the post-genomic era. In addition to genomics, transcriptome, and proteome analysis, dynamic analysis and means are essential to analyze transcription factor complexes and conformational changes in chromatin conformation. An innovative soft X-ray microscope operated in the water-window region is expected to be a very important and powerful tool. On the other hand, in combination with significant advances in observing technology, immunohistochemical techniques, fluorescent labeling techniques, and highly sensitive detection system, visualization techniques of biological macromolecules at the cellular level will progress with the help of advanced FRET spectroscopy.

Visualization of living cells by GFP gene cloning technology is an epoch-making new technology, with great impact on life sciences while bringing a new dimension in many fields such as cell lineage, the large conversion of cell position or movement, the study of embryology and morphology of the formation of functional networks, regenerative medicine, and neuroscience.

We have developed an entirely new technology that integrates visualization of biological macromolecules at the cellular level by time-resolved FRET techniques with coherent soft X-ray microscopy using our soft X-ray laser source to observe living cells under wet conditions. We are particularly focused on lipid rafts that act as a signaling platform. Thus the remarkable progress in highly sensitive detection system and observation technology such as functional genomics, visualization technology of molecular biological macromolecules, fluorescent techniques for a variety of proteins, and immunohistochemical techniques visualizes the dynamics of intracellular receptor proteins rarely expressed and furthermore enables visualization of various biochemical reactions in cells at single cell level and subcellular localization of functional proteins.

The specificity of the localization has not been completely clarified for a concentration of signaling molecules to lipid rafts rich in any kind of fat. A mixed state of various characteristics of lipid rafts has been considered.

By any cause or condition if a change in sphingolipid or cholesterol metabolism should occur, the quantitative changes of these fats alter the configuration in lipid rafts and localization of a variety of signaling substance changes will take place resulting in significant changes in signal transduction mediated through GPCR and other receptors. It is considered that this mechanism influences disease expression and development. The existence ratio of a lipid raft configuration will affect the ligand-receptor binding capacity, and results in a change in receptor-mediated signal transduction efficiency. Examination in detail of the signaling transduction changes along with the quantitative and qualitative changes of molecular components of lipid rafts due to disease and aging provides valuable information for prevention and treatment of various diseases. We will gain much knowledge about the relationship between drug action and intracellular localization and regulation of the receptor. It is expected that a detailed analysis of the cell receptor regulation will lead to the development of new drugs that target on GPCR localized in lipid rafts.

## Figures and Tables

**Table 1:** Intracellular proteins coupled with the C-terminus of GPCR.

**GPCRs**	**Intracellular proteins**
AT1	ATRAP, eNOS, JAK2
AT2	ATIP, PLZF
B2	eNOS
CL1	Shank1
D1	COPI, DRIP78
D_2_	CLIC6, GIPC, NCS-1
D_3_	CLIC6, GIPC
D_4_	CLIC6
ET_B_	eNOS
Frizzled	Dishevelled
GABA_B1/B2_	CREB2(ATF4)/ATFx.
mGlu_1a_	Tamalin
mGlu_1a,5a_	Homers1, 3
mGlu_4a,5a,5b,7a,7b,8a_	Filamin A
mGlu_7a,7b_	Gβγ/CaM, PICK-1, Syntenin
PTHI	14-3-3, 4.1G, NEHRF/EBP50, Tctex-1
P2Y_1_	NEHRF/EBP50
Rhodopsin	ARF4
5-HT_2A_	CIPP
5-HT_2A,2C_	ARIP/MAGI-2, PSD-95, SAP97
5-HT_2C_	Dlgh3/MPP-3, SAP102, Veli-3
β_1_	ARIP/MAGI-2, GIPC, PSD-95
β_2_	AKAP-250, NEHRF/EBP50
β_3_	Src
μ	Filamin A, Periplakin
κ	NEHRF/EBP50

AT1: Angiotensin AT1 receptors; AT2: Angiotensin AT2 receptors; B2: bradykinin B2 receptors; CL1: CIRL/latrophilin 1; D1: Dopamine D1; D2: Dopamine D2; D3: Dopamine D3; D4: Dopamine D4; ETB: Endothelin Receptor; Frizzled: Frizzled protein is a family of G protein-coupled receptor proteins that serve as receptors in the Wnt(catenation of Wg (wingless) and Int) signaling pathway and other signaling pathways; GABA: γ-aminobutyric acid; mGlu: metabotropic glutamate receptors; PTHI: parathyroid hormone; P2Y1: Purinergic receptor P2Y, G-protein coupled, 1; Rhodopsin: a membrane protein in the retina of the eye; 5-HT_2A_: serotonin 5HT2A(5-hydroxytryptamine) receptor; 5-HT_2C_: serotonin 5HT2C(5-hydroxytryptamine) receptor; β1: beta-adrenergic receptor 1; β2: beta-adrenergic receptor 2; β3: beta-adrenergic receptor 3; μ: Mu opioid receptor (MOP); κ: Kappa opioid receptor (KOP); 14-3-3: also known as YWHAS Protein (SFN protein); 4.1G: cytoskeletal protein; AKAP-250: A-kinase-anchoring protein 250; ARF4: ADP-ribosylation factor 4; ARIP: Activin receptor-interacting protein; MAGI-2: membrane-associated guanylate kinase inverted 2; ATRAP: angiotensin receptor-associated protein; ATIP: enigmatic Ang II type 2 (AT2)-interacting protein; CIPP: channel-interacting PDZ protein; CLIC6: Oryctolagus cuniculus chloride intracellular channel 6; COPI: coat protein I; Dishevelled: cytoplasmic phosphoprotein; Dlgh3: discs, large homolog 3 (Drosophila); MPP-3: membrane protein palmitoylated-3; DRIP78: dopamine receptor-interacting protein; eNOS endothelial nitric-oxide synthase; Filamin A: also known as ABP-280; Gβγ: Gbeta/gamma subunit of a trimeric G-protein; CaM: constitutively active mutants; GIPC: GAIP-interacting protein C-terminus; Homers1, 3: a member of the scaffolding Homer protein family; JAK2: janus kinase 2; NCS-1: neuronal calcium sensor 1, called frequenin in Drosophila; NEHRF: The Na(+)/H(+) exchange regulatory factor; EBP50: Ezrin/radixin/moesin (ERM) binding protein of 50 kDa; Periplakin: filament-binding protein; PICK-1: protein interacting with C kinase 1; PLZF: promyelocytic leukemia zinc finger; PSD-95: postsynaptic density-95; SAP: synapse-associated protein; Shank1: Somatostatin receptor-interacting protein, a family of postsynaptic proteins that function as part of the NMDA receptor-associated PSD-95 complex; Siah1A: seven in absentia homolog 1A; Src: tyrosine kinase; Syntenin: a PDZ protein that binds the cytoplasmic C-terminal FYA motif of the syndecans; Tamalin: 95 kDa postsynaptic density protein (PSD-95)/discs-large/ZO-1 (PDZ) domain-containing protein; Tctex-1: t-complex testis-expressed 1; Veli-3: Vertebrate lin-7 homolog 3.

**Table 2:** Decay time shortening of donor protein (CFP) by FRET mechanism.

**Specimens**	**decay time(ns) non-activated**	**decay time (ns) activated**
Cameleon	1.1	0.90 (82%)
GRIN1 + GoVenus	1.26	0.8 (64%)
GRIN2 + GoVenus	1.15	0.60 (52%)
